# Regulated Cell Death in Idiopathic Pulmonary Fibrosis

**DOI:** 10.1096/fj.202600494R

**Published:** 2026-05-02

**Authors:** Xiaoyue Pan, Liu Yang, Man Zhao, Yanlin Zhou, Cong Xia, Huibing Liu, Yuqi Wang, Hui Lian, Bin Li, Lan Wang, Guoying Yu

**Affiliations:** ^1^ State Key Laboratory of Cell Differentiation and Regulation, Henan International Joint Laboratory of Pulmonary Fibrosis, Henan Center for Outstanding Overseas Scientists of Organ Fibrosis, Pingyuan Lab, College of Life Science Henan Normal University Xinxiang China

**Keywords:** crosstalk, idiopathic pulmonary fibrosis, regulated cell death, therapeutic targets

## Abstract

Idiopathic pulmonary fibrosis (IPF) is a chronic, progressive interstitial lung disease characterized by deregulated cell death programs that drive epithelial injury, fibroblast activation, and irreversible tissue remodeling. Multiple regulated cell death (RCD) modalities, including apoptosis, autophagy, necrosis, ferroptosis, pyroptosis, and cuproptosis, are implicated in IPF pathogenesis across epithelial cells, fibroblasts, macrophages, and endothelial cells. Apoptosis leads to alveolar epithelial cell loss and fibrosis initiation, whereas autophagy modulates fibroblast proliferation and extracellular matrix turnover. Necrosis amplifies inflammation; ferroptosis promotes epithelial dysfunction through lipid peroxidation; and pyroptosis activates the inflammasome pathway. Emerging evidence links cuproptosis, a copper‐dependent death mode, to fibrotic remodeling. These pathways are interconnected: apoptosis and autophagy can shift within the same cell, and epithelial apoptosis may induce macrophage pyroptosis, amplifying the profibrotic cascade. Emerging evidence indicates that these RCD modalities are coordinated through shared stress signals and regulatory nodes. Therapeutically, targeting RCD offers promising opportunities, with Bcl‐2 inhibitors for apoptosis, mTOR inhibitors for autophagy, iron chelators for ferroptosis, and early interventions for pyroptosis and cuproptosis. Targeting shared regulatory mechanisms or combining pathway‐directed strategies may further enhance efficacy. By balancing cell death and survival, these strategies could attenuate inflammation, restrict fibroblast‐driven scarring, and restore repair capacity. This review underscores the complexity and crosstalk of RCD in IPF, and proposes a conceptual framework for their coordinated regulation, highlighting its potential for therapeutic innovation.

## Graphical abstract

This graphical abstract illustrates regulated cell death (RCD) across key pulmonary cell types—including alveolar epithelial cells, fibroblasts, macrophages, and endothelial cells—modulated through a dynamic death modulation network within the IPF microenvironment. These processes lead to epithelial loss, inflammation, extracellular matrix deposition, and vascular remodeling, ultimately driving progressive fibrosis.

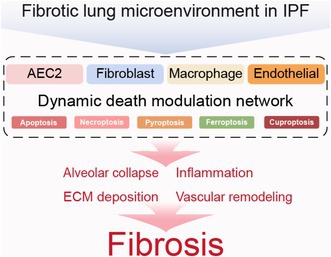

## 1 Introduction

Idiopathic pulmonary fibrosis (IPF), first noted as diffuse interstitial fibrosis in the 1930s and later defined as a distinct entity in the ATS/ERS consensus statement [1], is a progressive and fatal lung disorder characterized by irreversible scarring that compromises gas exchange, induces persistent dyspnea, and ultimately results in respiratory failure. Epidemiologic data indicate that the global incidence ranges from 0.2 to 17.4 per 100 000 individuals annually, with a prevalence of about 51.7 per 100 000 (95% CI: 24.5–78.8), and an upward trend is evident in several regions, particularly in aging populations [2]. Median survival remains limited to only 3–4 years following diagnosis [3]. Current antifibrotic drugs, including pirfenidone and nintedanib, slow but do not halt disease progression. The scarcity of effective interventions underscores the need to investigate novel mechanisms, signaling networks, and molecular drivers that sustain fibrosis and may provide therapeutic opportunities.

Histologically, IPF is characterized by the usual interstitial pneumonia (UIP) pattern, featuring excessive extracellular matrix (ECM) deposition, mainly collagen, forming fibrotic foci interspersed with relatively preserved parenchyma [4, 5]. These lesions disrupt normal lung architecture through complex interactions among alveolar epithelial cells (AECs), fibroblasts, macrophages, and endothelial cells (ECs). Repeated AEC injury initiates fibrosis via transforming growth factor‐β (TGF‐β) release, which drives fibroblast activation and myofibroblast differentiation, enhancing collagen synthesis and ECM accumulation. Persistent crosstalk with immune cells, particularly macrophages, sustains a profibrotic and proinflammatory milieu [6]. This self‐perpetuating cycle of epithelial injury, fibroblast activation, and matrix remodeling leads to irreversible scarring, emphasizing the need for deeper mechanistic insight to identify novel therapeutic targets in IPF.

**FIGURE 1:**
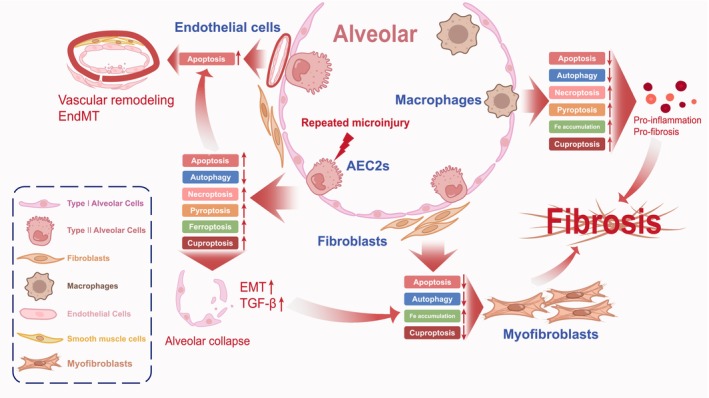
Cell‐type–specific dysregulation of regulated cell death pathways drives the progression of IPF. Schematic overview illustrating how distinct forms of regulated cell death in major pulmonary cell types contribute to alveolar collapse, chronic inflammation, and fibrotic remodeling in IPF. Within the alveolar unit, type II alveolar epithelial cells (AEC2s) are repeatedly exposed to microinjury, leading to upregulated apoptosis, necroptosis, ferroptosis, pyroptosis, and cuproptosis, alongside suppressed autophagy. These cumulative perturbations cause alveolar collapse, promote epithelial–mesenchymal transition (EMT), and drive the release of TGF‐β, initiating fibrogenic signaling. In contrast, fibroblasts exhibit downregulated apoptosis and autophagy, coupled with iron accumulation and decreased cuproptosis, which collectively enhance their survival and differentiation into myofibroblasts, the principal effector cells responsible for excessive extracellular matrix (ECM) deposition and tissue scarring. Alveolar macrophages show reduced apoptotic and autophagic turnover but heightened inflammatory cell death modalities—necroptosis, ferroptosis, pyroptosis, and cuproptosis—leading to persistent pro‐inflammatory signaling and a maladaptive transition toward a pro‐fibrotic, tissue‐remodeling phenotype. Through sustained secretion of pro‐fibrotic factors, these macrophages perpetuate fibroblast activation and chronic injury. Endothelial cells undergo enhanced apoptosis, contributing to vascular rarefaction, remodeling, and Endothelial‐to‐Mesenchymal Transition (EndMT), further amplifying fibroblast accumulation and microvascular dysfunction. Together, these cell‐specific imbalances in regulated cell death form a multicellular pathogenic network that underlies the irreversible progression of IPF.

Cell death is a central determinant of organ function and tissue homeostasis, selectively eliminating damaged or superfluous cells through tightly regulated mechanisms that preserve development, repair, and physiological equilibrium [7]. In IPF, AECs are particularly susceptible to injury‐induced death, whereas fibroblasts demonstrate resistance to cell death alongside enhanced proliferative potential, collectively driving fibrosis initiation and progression [8]. Classical investigations of cell death in IPF have focused on apoptosis, autophagy, pyroptosis, necroptosis, and ferroptosis, whereas emerging studies highlight cuproptosis as an additional relevant pathway [9]. These death programs exhibit cell type‐specific features in epithelial, fibroblast, macrophage, and endothelial compartments, and act in concert rather than isolation to shape the fibrotic microenvironment (Figure 1). Increasing evidence suggests that these processes are coordinated through shared stress signals and interconnected regulatory mechanisms, rather than functioning as independent pathways. While epithelial cell death—particularly apoptosis—is widely recognized as a central driver of IPF pathogenesis, cell death processes in fibroblasts and macrophages may also contribute to disease progression in a context‐dependent manner, particularly through their roles in shaping the fibrotic microenvironment.

Given the limited therapeutic options and poor prognosis associated with IPF, there is an urgent need to delineate the cellular and molecular mechanisms underlying disease progression. In this review, we provide a comprehensive synthesis of diverse cell death mechanisms—including apoptosis, autophagy, necroptosis, pyroptosis, ferroptosis, and cuproptosis—emphasizing their functional roles in AECs, fibroblasts, macrophages, and ECs. We outline therapeutic opportunities for each modality and examine the crosstalk that shapes fibrotic pathogenesis. In particular, we aim to integrate these pathways within a conceptual framework that considers their coordinated regulation, shared stress inputs, and context‐dependent activation. By integrating mechanistic and therapeutic insights, this review aims to inform future research and advance IPF interventions.

## 2 Apoptosis

**FIGURE 2:**
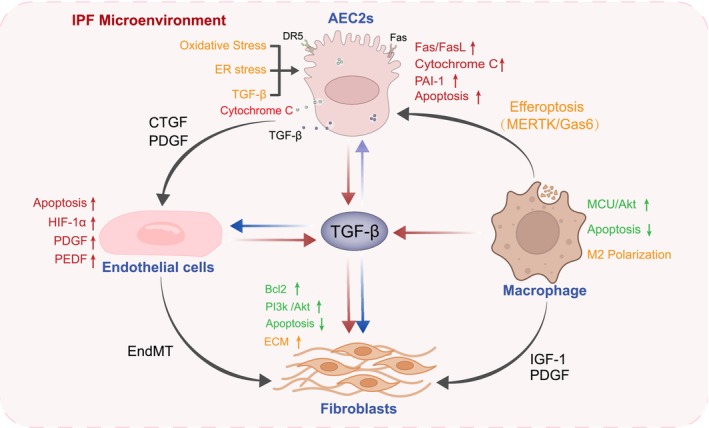
Schematic representation of the mechanisms underlying apoptosis dysregulation in AEC2s, endothelial cells, fibroblasts, and macrophages in IPF. This figure depicts the apoptotic features and their interactive network among AEC2, ECs, fibroblasts, and macrophages in IPF. AEC2s undergo prominent apoptosis induced by oxidative stress, endoplasmic reticulum (ER) stress, and other damaging stimuli, leading to the release of pro‐fibrotic mediators such as transforming growth factor‐β (TGF‐β) and connective tissue growth factor (CTGF). ECs initially exhibit reduced apoptosis and enhanced proliferation under the anti‐apoptotic influence of vascular endothelial growth factor (VEGF), facilitating neovascular formation. In later stages, pigment epithelium‐derived factor (PEDF) becomes dominant, promoting EC apoptosis and vascular loss while inducing EndMT in surviving ECs, thereby expanding fibroblast‐like populations and aggravating hypoxia and fibrosis. Macrophages are predominantly polarized toward the M2 phenotype, exhibiting anti‐apoptotic properties. Through MERTK/Gas6‐mediated efferocytosis of apoptotic AEC2 debris, they release TGF‐β, which amplifies fibrogenic signaling. Fibroblasts display strong anti‐apoptotic characteristics mediated through the PI3K/Akt pathway with upregulated Bcl‐2 expression and further reinforced by macrophage‐derived factors such as platelet‐derived growth factor (PDGF) and insulin‐like growth factor‐1 (IGF‐1), resulting in enhanced proliferation and ECM deposition. Together, this figure highlights how the imbalance between pro‐apoptotic and anti‐apoptotic mechanisms among different cell types orchestrates the progressive fibrotic remodeling observed in IPF.

Apoptosis, a form of programmed cell death, is essential for tissue development and homeostasis. It is orchestrated through coordinated regulation of gene expression, cell cycle dynamics, and caspase activation to eliminate damaged or superfluous cells [10]. Apoptotic responses can be triggered by diverse stimuli, with susceptibility and execution efficiency varying across cell types and physiological contexts. Variations in cellular responsiveness, including resistance to apoptosis, are central to disease pathogenesis, such as in IPF, and represent critical considerations for developing targeted therapeutic strategies [11]. The mechanisms of apoptosis dysregulation across different pulmonary cell types in IPF are summarized in Figure 2.

### 2.1 Apoptosis in Epithelial Cells

In IPF, the so‐called “apoptotic paradox” represents a central pathological hallmark. Substantial evidence indicates that apoptosis is markedly increased in AECs—particularly in type II alveolar epithelial cells (AEC2s)—whereas fibroblasts exhibit reduced sensitivity to apoptotic stimuli [12]. AEC2s are responsible for synthesizing and secreting pulmonary surfactant [13], and their loss not only disrupts alveolar barrier integrity but also impairs epithelial regenerative capacity, thereby contributing to structural remodeling imbalance. This paradox reflects a fundamental imbalance in cell fate, where epithelial vulnerability coexists with mesenchymal persistence, impairing normal tissue repair in IPF. In lung tissues from IPF patients, apoptosis‐related proteins such as Bax and Caspase‐3 are markedly upregulated, indicating sustained activation of apoptotic pathways.

Apoptosis of AEC2s is driven by multiple interconnected mechanisms, which are better understood as components of a unified epithelial stress network converging on apoptotic execution. Excessive activation of the Fas/FasL signaling pathway induces extrinsic, caspase‐dependent apoptosis in AEC2s [14]. This process is further potentiated by TGF‐β, which upregulates Fas receptor expression and sensitizes cells to FasL, thereby amplifying apoptotic signaling [15]. Persistent ER stress and UPR activation [5, 16], together with mitochondrial DNA damage and oxidative stress, disrupt AEC2 homeostasis and induce apoptosis via p53 and caspase pathways [17]. These stressors—including proteostatic imbalance, mitochondrial dysfunction, and redox disturbance—are interconnected and collectively lower the apoptotic threshold of AEC2s. Under excessive or sustained stress, these factors may also trigger ferroptosis or pyroptosis [18] through interacting positive feedback, yet in IPF, apoptosis remains the predominant outcome due to dominant mitochondrial dysfunction and sustained caspase activation; this is likely due to mitochondrial dysfunction and sustained caspase activation acting as convergent execution pathways. Furthermore, telomere shortening and reduced telomerase activity in senescent AEC2s diminish regenerative capacity and heighten apoptotic susceptibility [19]. Senescence‐associated secretory phenotype (SASP) factors subsequently intensify local inflammation and fibrogenic signaling [5].

Apoptosis of AEC2s represents a pivotal early event in IPF. Loss of surfactant homeostasis destabilizes alveolar architecture, leading to collapse, septal distortion, and compensatory dilatation of alveolar ducts [8, 20]. These structural abnormalities create a microenvironment that favors fibroblast activation as the tissue attempts to restore alveolar integrity. In addition to surfactant synthesis, AEC2s release antifibrotic mediators such as prostaglandin E2 (PGE2), which suppress fibroblast proliferation and promote matrix turnover through plasminogen activators and matrix metalloproteinases [21]. Extensive AEC2 apoptosis abolishes these protective functions, thereby enabling uncontrolled fibroblast expansion and ECM deposition.

Increasing attention has focused on efferocytosis—the macrophage‐mediated clearance of apoptotic cells [22]. Apoptosis produces membrane‐bound apoptotic bodies that are recognized and engulfed by phagocytes. In a mouse model, apoptosis was induced in GFP‐labeled AEC2s using ultraviolet radiation, after which alveolar macrophages (AMs) efficiently engulfed the apoptotic cells [23]. This uptake reprograms macrophages toward a profibrotic phenotype, marked by increased arginase and TGF‐β expression with reduced iNOS. These findings indicate that AEC2 apoptosis promotes macrophage profibrotic polarization via efferocytosis, rather than being a passive consequence of epithelial injury. Subsequent in vivo studies confirmed that dysregulated efferocytosis contributes to IPF progression, highlighting its potential as a therapeutic target for restoring macrophage homeostasis. Thus, efferocytosis may represent a key interface linking epithelial cell death to immune‐driven fibrogenesis, indicating that the impact of apoptosis extends beyond cell loss to downstream microenvironmental reprogramming.

### 2.2 Apoptosis in Fibroblasts

In contrast to the enhanced apoptosis observed in AEC2s, fibroblasts within the IPF microenvironment display marked resistance to apoptosis. Early investigations demonstrated that fibroblasts derived from IPF lungs exhibit reduced susceptibility to Fas ligand (FasL)–induced apoptosis compared with normal lung fibroblasts [24]. This apoptotic resistance has been associated with diminished cell surface Fas expression and elevated levels of soluble Fas, both of which attenuate Fas‐mediated death signaling. Within fibrotic lesions, upregulation of Decoy Receptor 3 (DcR3) and nuclear factor of activated T‐cells cytoplasmic 1 (NFATc1) inhibits Fas–FasL–induced apoptosis through direct FasL binding [25]. These findings indicate that fibroblast apoptosis resistance is partly determined at the level of upstream death signal availability and receptor engagement. Besides, accumulating evidence suggests that apoptotic outcomes cannot be fully explained by upstream signaling alone, but also depend on how these signals are decoded within cells. Reduced cyclooxygenase‐2 (COX‐2)–derived prostaglandin E_2_ (PGE_2_) has been shown to exacerbate epithelial apoptosis while conferring apoptosis resistance to fibroblasts [21], indicating that distinct cell types decode identical cues through different intracellular signaling frameworks. A similar phenomenon is observed within the same cell type. Interleukin‐6 (IL‐6), which enhances Bax expression and promotes Fas‐induced apoptosis in normal fibroblasts, induces Bcl‐2 expression and suppresses apoptosis in IPF fibroblasts [26], due to the abnormal activation of ERK signaling in IPF fibroblasts. Thus, even within fibroblasts, state‐dependent signaling rewiring can drive opposite cell fate outcomes in response to the same stimulus.

Resistance of IPF fibroblasts to apoptosis extends beyond death receptor pathways to include intrinsic mitochondrial mechanisms. Under normal conditions, mitochondrial apoptosis is initiated by intracellular stressors that trigger cytochrome c release, leading to apoptosome assembly, caspase activation, and cell death. In IPF fibroblasts, mitochondrial dysfunction is well documented [27], characterized by elevated mitochondrial superoxide, DNA damage, fragmentation of mitochondrial networks, and reduced cytochrome c content. These alterations promote a senescent phenotype while diminishing sensitivity to apoptosis induced by mitomycin C [28]. Furthermore, fibroblasts from fibrotic lungs exhibit sustained activation of the mTOR pathway, which further suppresses apoptotic susceptibility [29]. Collectively, these mechanisms underlie apoptosis resistance in IPF fibroblasts. Notably, key regulatory nodes such as Bcl‐2 signaling, mitochondrial remodeling, and mTOR activation also govern multiple cell death‐related processes, including autophagy, metabolic regulation, and oxidative stress, suggesting that these shared regulatory nodes may confer a broader resistance to multiple forms of regulated cell death (RCD)—including necroptosis and ferroptosis—rather than selectively inhibiting apoptosis alone.

### 2.3 Apoptosis in Macrophages

Macrophages not only mediate the profibrotic consequences of AEC2 apoptosis [30] but their own apoptosis has also been implicated in the progression of IPF [31]. Pulmonary macrophages comprise two principal subpopulations: AMs, residing within the alveolar spaces, and interstitial macrophages (IMs), which occupy the lung parenchyma. AMs function as frontline immune effectors and exhibit both proinflammatory and anti‐inflammatory activities, whereas IMs are critical for maintaining immune homeostasis and promoting tolerance to nonpathogenic antigens [32]. Both subsets exert substantial influence on the initiation and progression of pulmonary fibrosis. In particular, AMs are recognized as a major source of profibrotic mediators that orchestrate fibroblast activation and extracellular matrix deposition. Consequently, recent studies have focused on elucidating the cell death programs of AMs and their contributions to fibrogenesis [33].

As early as 2002, macrophages preconditioned for apoptosis in vitro were instilled into rat lungs, resulting in macrophage accumulation, enhanced cell death, collagen deposition, and fibrotic lesions [34]. In contrast, a 2012 clinical study reported that AMs isolated from bronchoalveolar lavage fluid (BALF) of IPF patients exhibited reduced apoptosis compared with healthy controls [35]. Rather than a discrepancy, these findings likely reflect stage‐dependent roles of macrophage apoptosis, with early injury‐associated death amplifying damage, whereas chronic disease is characterized by apoptosis resistance and macrophage persistence. Subsequent studies confirmed that macrophages in IPF lungs acquire apoptosis resistance, a key feature driving their profibrotic activation [36, 37].

The persistence of AMs with reduced apoptotic susceptibility is thought to drive fibrosis by promoting polarization toward a profibrotic state [38]. AMs polarize into classically activated M1 or alternatively activated M2 phenotypes. M1 macrophages secrete proinflammatory cytokines that control infection, whereas M2 macrophages exhibit anti‐inflammatory and profibrotic functions through mediators such as TGF‐β [39, 40, 41]. In IPF, macrophages with an apoptosis‐resistant, M2‐like phenotype are markedly expanded. Notably, single‐cell RNA sequencing studies suggest that this persistence reflects the expansion of specific profibrotic macrophage subsets, rather than a uniform shift across the entire macrophage population [42], which highlights the need to define cell death characteristics at the level of specific macrophage subsets, rather than treating macrophages as a homogeneous population.

Mechanistically, macrophage apoptosis resistance appears to arise from the integration of mitochondrial regulation, metabolic reprogramming, and survival signaling pathways. At the mitochondrial level, enhanced mitophagy mediated by Akt1 activation has been shown to underlie apoptotic resistance and promote sustained TGF‐β secretion, thereby driving myofibroblast differentiation and fibrotic progression [36]. In parallel, upregulation of anti‐apoptotic proteins further reinforces this survival advantage; Cpt1a, a key enzyme in fatty acid β‐oxidation, interacts with the BH3 domain of Bcl‐2 to stabilize its mitochondrial localization under the control of the mitochondrial calcium uniporter (MCU) [43]. At the metabolic interface, elevated sphingomyelin (SM) and phosphatidylcholine (PC) in fibrotic lungs activate TREM2–AKT/ERK signaling in monocyte‐derived macrophages, inducing Bcl‐xL and Mcl‐1 to inhibit apoptosis; loss of TREM2 abrogates this protection, linking lipid remodeling to macrophage persistence [44]. Notably, these distinct upstream signals converge on shared survival pathways, particularly the Akt and Bcl‐2 family axes, suggesting the presence of a coordinated regulatory network rather than isolated signaling events. Beyond apoptosis, coordinated changes in mitochondrial function, metabolism, and survival signaling may reshape macrophage death regulation, with emerging evidence indicating crosstalk among apoptosis, autophagy, and inflammatory cell death in pulmonary fibrosis [31], suggesting a broader rewiring of death susceptibility.

Macrophage apoptosis resistance occurs not only in bleomycin models but also in asbestos‐induced fibrosis. NADPH oxidase 4 (NOX4) activates Akt, which phosphorylates BAD (Ser136 in mice; Ser99 in humans), preventing its interaction with Bcl‐2. This blockade enables macrophages to evade apoptosis and maintain profibrotic activity [45].

### 2.4 Apoptosis in Endothelial Cells

ECs, sharing the alveolar basement membrane with AECs, form a key part of the air–blood barrier. AEC damage triggers TGF‐β and reactive oxygen species (ROS) release, creating local hypoxia that stabilizes HIF‐1α in AECs and ECs. HIF‐1α induces VEGF expression and activates PI3K/Akt signaling, promoting ECs' survival and early tissue repair [46, 47].

As lesions progress (3–7 days in animal models, equivalent to months in humans), ECs activated by AEC‐derived TGF‐β and IL‐1β upregulate ICAM‐1 and VCAM‐1, promoting macrophage recruitment and inflammation, while widespread apoptosis is still absent [48, 49].

Persistent AEC injury and macrophage activation sustain TGF‐β release, which exerts dose‐dependent effects on ECs: low levels promote ECs angiogenesis, whereas high levels inhibit proliferation and induce apoptosis [50]. Excessive TGF‐β activates Smad2/3 signaling, triggering ER stress, CHOP induction, and caspase‐3/9–mediated mitochondrial apoptosis. This response is amplified by pigment epithelium–derived factor (PEDF). In IPF, overall PEDF levels are elevated, primarily induced by TGF‐β1 stimulation and hypoxia/ROS stress. Initially, PEDF may restrain aberrant angiogenesis and fibroblast activation, but chronic stimulation renders it pro‐apoptotic. Predominantly produced by fibroblasts, myofibroblasts, and some epithelial cells [51], PEDF suppresses EC migration, tube formation, and VEGF expression, while inducing apoptosis via Fas/FasL signaling. It primarily targets immature neovessels, whereas mature vessels remain relatively resistant, suggesting a selective vascular “pruning” that may ultimately limit fibrosis [52, 53].

As the disease advances into the mid‐to‐late stages, VEGF expression declines from its initial elevation, largely due to TGF‐β inhibition and PEDF antagonism. This reduction diminishes VEGF‐mediated anti‐apoptotic protection of ECs, thereby tipping the balance toward endothelial cell death [54].

Endothelial apoptosis does not occur in isolation. Apoptotic ECs release damage‐associated molecular patterns (DAMPs), which enhance TGF‐β signaling and induce endothelial‐to‐mesenchymal transition (EndMT) in surviving ECs, marked by loss of VE‐cadherin and upregulation of α‐SMA and collagen I, functioning both as an apoptosis escape mechanism and as a source of pro‐fibrotic cells [55, 56]. EC loss due to apoptosis causes microvascular rarefaction and permeability increases, aggravating hypoxia and stabilizing HIF‐1α. Under high TGF‐β and PEDF with low VEGF, hypoxia signaling becomes pro‐fibrotic, sustaining inflammation and ECM deposition, ultimately driving irreversible vascular remodeling and fibrosis [12, 57].

### 2.5 Potential Therapeutic Strategies Related to Apoptosis

Given the above, targeting apoptosis represents a promising therapeutic strategy for IPF. Inhibition of the Fas–FasL pathway directly interrupts death signaling in AEC2s, markedly attenuating bleomycin‐induced fibrosis in animal models, though potential immunosuppressive risks limit clinical translation. At the metabolic level, suppression of glutathione‐S‐transferase P (GSTP) mitigates Fas S‐glutathionylation and AEC2 apoptosis, with both genetic deficiency and pharmacologic inhibition conferring robust antifibrotic effects and favorable safety profiles [58]. At the transcriptional tier, miR‐29c overexpression enhances epithelial proliferation and protects against apoptosis induced by bleomycin or ER stress via Foxo3a downregulation, thereby restoring epithelial integrity and restraining fibrotic remodeling [59].

In fibroblasts, restoring apoptotic susceptibility offers another antifibrotic strategy. Inhibition of X‐linked inhibitor of apoptosis (XIAP) suppresses fibrogenesis by reactivating cell death pathways [60]. Histone deacetylase inhibition with suberoylanilide hydroxamic acid (SAHA), a clinically approved anticancer agent, promotes myofibroblast apoptosis via Bak upregulation and Bcl‐xL suppression, improving fibrosis and lung function in bleomycin models [61]. Similarly, BH3 mimetics such as ABT‐263 selectively induce apoptosis in α‐SMA^+^/Bcl‐2^+^ fibroblasts, effectively reversing fibrosis in bleomycin and silica models [62].

Targeting apoptosis in profibrotic macrophages has also emerged as a potential therapeutic strategy. Inhibition of Cpt1a and fatty acid oxidation (FAO) has been shown to induce macrophage apoptosis [43], as have interventions aimed at modulating ER stress and unfolded protein response (UPR) pathways, including Grp78 and CHOP [63]. Notably, although ABT‐199 increases apoptosis in AEC2s, established fibrosis can still be reversed [43], suggesting that AEC2s' apoptosis alone is insufficient to drive fibrosis without contributions from other cell types, particularly monocyte‐derived macrophages. Soluble TREM2 (sTREM2) acts as a decoy receptor that neutralizes SM, and both sTREM2 and TREM2‐blocking antibodies significantly attenuate pulmonary fibrosis, even with delayed treatment, underscoring their therapeutic promise. Selectively eliminating profibrotic macrophages while preserving reparative subsets remains critical for achieving antifibrotic efficacy with minimal systemic risk.

Although endothelial apoptosis contributes to capillary rarefaction and tissue remodeling, no validated endothelial‐specific therapies currently exist. Key regulators such as VEGF and TGF‐β influence endothelial survival but act broadly across cell types, complicating direct intervention. VEGF overexpression can restore vascular integrity yet may aggravate fibrosis through nonspecific delivery, fibroblast activation, and ectopic angiogenesis. Approved antifibrotic agents like pirfenidone and nintedanib mainly suppress systemic profibrotic signaling (TGF‐β, PDGF, FGF) rather than targeting endothelium directly. Thus, current IPF management focuses on preserving vascular function and improving the microenvironment. Future discovery of endothelial‐specific molecular checkpoints may enable safer, targeted modulation of apoptosis and angiogenesis in fibrosis. A summary of apoptosis‐related therapeutic strategies, experimental models, and targets in IPF is provided in Table 1.

**TABLE 1:** Experimental models and therapeutic strategies targeting apoptosis in IPF.

Experimental model	Cell type	Therapeutic approach	Target/Drug	References
Bleomycin or TGF‐β overexpression mouse model	AEC2	Inhibition of GSTP reduces FAS S‐glutathionylation and apoptosis	GSTP knockout or GSTP inhibitors	McMillan et al. (2016)
Bleomycin‐induced mouse model	AEC2	miRNA‐based intervention enhances proliferation and protects against apoptosis	miR‐29c (targeting Foxo3a)	Xie et al. (2017)
Primary fibroblasts (IPF/non‐IPF patients)	Fibroblasts	Restoration of apoptosis sensitivity via XIAP inhibition	XIAP inhibitors	Ajayi et al. (2013)
Bleomycin‐induced mouse model	Fibroblasts	Induction of apoptosis through HDAC inhibition	SAHA (upregulates Bak, downregulates Bcl‐xL)	Sanders et al. (2014)
Bleomycin‐induced mouse model	Fibroblasts	BH3 mimetics induce apoptosis and alleviate fibrosis	ABT‐263	Cooley et al. (2022)
Bleomycin and silica‐induced mouse models	AEC2/Macrophages	Bcl2 inhibitor, induce apoptosis and alleviate fibrosis	ABT‐199 (increased AEC2 apoptosis, but fibrosis still reversed)	Gu et al. (2021)
Bleomycin and silica‐induced mouse models	Macrophages	Metabolic intervention to induce apoptosis	Inhibition of Cpt1a and fatty acid oxidation (FAO)	Gu et al. (2021)
Bleomycin‐induced mouse model	Macrophages	Targeting ER stress/UPR pathways to promote apoptosis	Grp78, CHOP	Ayaub et al. (2016)

## 3 Autophagy

Autophagy, an evolutionarily conserved program essential for eukaryotic cell survival, mediates the degradation of proteins, aggregates, damaged organelles, and exogenous particles, thereby mitigating cellular stress, limiting injury, and regulating senescence and differentiation [64]. Although primarily cytoprotective, dysregulated autophagy within the IPF microenvironment—persistent deficiency or excessive activation—can paradoxically promote AEC death and alter fibroblast apoptosis resistance, converting a normally protective mechanism into a profibrotic pathway [65]. Autophagy and apoptosis are further interconnected through shared regulatory nodes, including the Beclin‐1/Bcl‐2 axis and mitochondrial networks, which collectively govern cell fate decisions [66]. Through its control of iron homeostasis, lipid peroxidation, and inflammasome activity, autophagy is also positioned to modulate susceptibility to other regulated cell death programs, most notably ferroptosis [67, 68] and, to a lesser extent, pyroptosis [69, 70], thereby integrating multiple death pathways. Despite consistent evidence of reduced autophagy in IPF lung tissues, the net functional consequences for fibrosis progression and the potential for therapeutic modulation remain incompletely defined.

### 3.1 Autophagy in Epithelial Cells

Autophagy functions as a fundamental stress‐adaptive mechanism, and maintenance of autophagic flux is critical for epithelial homeostasis. In IPF and experimental models, epithelial integrity is compromised in parallel with suppressed autophagy. Hyperactivation of the mTOR pathway has emerged as a key suppressor of epithelial autophagy. In an AEC‐specific Tsc1 knockout model, mTOR activation inhibited autophagic flux, promoted apoptosis, and aggravated bleomycin‐induced fibrosis—effects reversed by rapamycin through restoration of autophagic activity [71]. Beclin1, a key initiator of autophagosome formation, is reduced within fibrotic regions of IPF lungs, contributing to impaired autophagic initiation [72]. This impairment has been partly associated with reduced expression of the deubiquitinase USP13, which supports autophagy via interaction with and deubiquitination of Beclin1. USP13 downregulation observed in IPF and bleomycin models contributes to impaired autophagic activity and increased fibrotic susceptibility in a Beclin1‐dependent manner [73]. Furthermore, disruption of ATG4B impairs autophagosome maturation, leading to enhanced epithelial apoptosis and collagen accumulation [74]. Together, these findings indicate that autophagy is disrupted at multiple levels, including initiation, regulation, and autophagosome maturation, ultimately leading to impaired autophagic flux. Inhibition of autophagy in AEC2s also triggers epithelial–mesenchymal transition and paracrine fibroblast activation through Snail2‐mediated signaling [75]. Such disruptions in autophagic regulation emerge as a critical constraint on epithelial stress adaptation, shifting cell fate toward the engagement of multiple downstream RCD pathways under persistent injury. However, the precise thresholds and temporal dynamics by which defective autophagy drives the transition toward distinct cell death programs remain poorly defined.

Autophagy in bronchial epithelial cells also plays an essential role in IPF and is closely linked to cellular aging. Inhibition of autophagy accelerates ER stress–induced senescence in human bronchial epithelial cells [72]. Taken together, these observations point toward impaired epithelial autophagy as a central pathogenic mechanism, suggesting that targeted enhancement of autophagic pathways could emerge as a promising therapeutic axis to halt or even reverse fibrotic remodeling in the lung.

### 3.2 Autophagy in Fibroblasts

In primary fibroblasts isolated from IPF lungs, a similar reduction in Beclin1 expression has been observed, indicating diminished autophagic flux. In contrast to epithelial cells, where impaired autophagy promotes cell loss, defective autophagy in fibroblasts primarily contributes to their persistence and resistance to cell death. This defect may arise from interactions between the anti‐apoptotic protein Bcl‐2 and Beclin1, which coordinate cellular survival and death pathways. Bcl‐2 can directly inhibit starvation‐induced autophagy through binding to Beclin1 [76]. Notably, IPF fibroblasts exhibit apoptosis resistance accompanied by elevated Bcl‐2 expression, suggesting that autophagy suppression and apoptosis resistance are not independent events, but part of a coordinated survival program in IPF fibroblasts. This altered balance likely sustains inhibition of Beclin1‐dependent autophagy and autophagic cell death. When normal lung fibroblasts are cultured on type I collagen‐rich matrices, autophagy is initially induced; in contrast, IPF fibroblasts maintain low autophagic activity under the same conditions, suggesting desensitization to collagen‐induced autophagy and cell death, which may facilitate fibrotic progression [77]. Mechanistically, this phenomenon is attributed to Akt hyperactivation in IPF fibroblasts, leading to reduced transcriptional activity of FoxO3a, a key regulator of LC3B expression. Furthermore, treatment with the autophagy inhibitor chloroquine (CQ) or LC3B siRNA significantly increases cell death in control fibroblasts compared with collagen‐treated IPF fibroblasts, indicating that reduced autophagy in IPF fibroblasts confers resistance to autophagy inhibition. Inadequate autophagy also plays a central role in myofibroblast differentiation; silencing LC3 or ATG5 via siRNA markedly enhances TGF‐β‐induced myofibroblast differentiation and type I collagen expression [72]. Collectively, these findings suggest that IPF fibroblasts maintain low autophagic activity through altered sensing mechanisms, which promotes their survival under diverse stimuli and facilitates differentiation into myofibroblasts, thereby accelerating disease progression. However, it remains unclear whether reduced autophagy in IPF fibroblasts reflects a global defect in autophagic flux or involves more selective alterations in organelle‐specific quality control pathways, such as mitophagy, which have not been well defined in fibroblasts in the context of IPF. Moreover, beyond regulating survival and differentiation, whether altered autophagy in IPF fibroblasts also reshapes their profibrotic secretory function remains largely unexplored.

### 3.3 Autophagy in Macrophages

In AMs, autophagy and mitophagy critically influence their polarization and fate, exhibiting distinct stage‐ and cell type–dependent dual effects. Conditional deletion of Atg7 or Atg5 in mice leads to spontaneous sterile pneumonia even under noninfectious conditions, characterized by sustained activation of the NLRP3 inflammasome and elevated secretion of IL‐1β and IL‐18, which in turn trigger IL‐17–TGF‐β cascades, forming an inflammation–fibrosis feedback loop, suggesting that defective autophagy in macrophages during early IPF may contribute to inflammatory amplification and initiate fibrotic remodeling [78]. At this phase, M1‐polarized macrophages exhibit mitochondrial dysfunction, ROS and mtDNA leakage, and sustained oxidative stress due to impaired autophagic clearance—priming TGF‐β activation and fibroblast recruitment.

During the progression phase, macrophage autophagy shifts toward enhanced selective mitophagy. IPF macrophages display elevated Akt1 phosphorylation, activating PINK1/Parkin‐dependent mitophagy driven by mitochondrial ROS, which facilitates Parkin recruitment and LC3‐II–positive autophagosome formation. Akt1‐mediated mitophagy maintains mitochondrial quality and metabolism while conferring anti‐apoptotic capacity and persistent TGF‐β1 release, sustaining fibrogenesis. Conversely, Park2^−^/^−^ or Akt1‐deficient macrophages undergo apoptosis and exhibit antifibrotic phenotypes, indicating that moderate mitophagy inhibition may halt fibrosis [36]. These findings suggest that mitophagy serves as a critical checkpoint linking mitochondrial stress adaptation to macrophage survival and profibrotic function.

In advanced disease, macrophages adopt an M2 pro‐fibrotic phenotype. miR‐33, upregulated in IPF macrophages, suppresses PGC‐1α and SIRT3, restricting autophagy and mitochondrial biogenesis. Its deletion restores the AMPK–PGC‐1α–SIRT3 pathway, enhances autophagy, and promotes a pro‐repair phenotype [79]. It is therefore proposed that profibrotic macrophages rely on elevated mitophagy to gain apoptosis resistance and sustain TGF‐β signaling, whereas low autophagic flux helps maintain their fibrogenic identity. However, it remains unclear whether reduced bulk autophagy and enhanced mitophagy occur within the same macrophage, arise from distinct subsets, or reflect stage‐dependent shifts, which will require resolution at the single‐cell level with temporal context to distinguish these possibilities. In addition, TGF‐β has been shown to induce autophagy via CCR8 upregulation, driving macrophage‐to‐myofibroblast transition (MMT) and suggesting a role for autophagy in structural remodeling during late‐stage disease [80].

### 3.4 Potential Therapeutic Strategies Related to Autophagy

In IPF, reduced autophagy drives apoptosis in AECs while enhancing fibroblast survival. This disparity reflects distinct “death thresholds”: epithelial cells require autophagy to clear damaged mitochondria, whereas fibroblasts, with elevated anti‐apoptotic protein levels, are inherently resistant, and loss of autophagy further stabilizes this resistance. Restoration of autophagy could simultaneously repair the epithelial barrier and limit fibroblast persistence, representing a promising therapeutic approach. PLAC8, downregulated in IPF, promotes p53 degradation via the VCP–UFD1–NPLOC4 complex, thereby restoring autophagy, reducing AEC2 apoptosis, and alleviating bleomycin‐induced fibrosis. These findings identify the PLAC8–p53–autophagy axis as a potential therapeutic target [81]. In bleomycin‐induced models, rapamycin shows therapeutic benefit only when administered early; delayed treatment with rapamycin or sirolimus yields no significant effect [71]. The loss of efficacy likely reflects established myofibroblast differentiation and matrix stiffening, which render myofibroblasts resistant to autophagic modulation. Nintedanib, a small molecule approved for the treatment of IPF, targets multiple tyrosine kinases, including fibroblast growth factor receptors (FGFRs), vascular endothelial growth factor receptors (VEGFRs), and platelet‐derived growth factor receptors (PDGFRs). Notably, nintedanib has also been reported to modulate autophagy by inducing a form of noncanonical autophagy that is Beclin1‐dependent but Atg7‐independent [82]. In a bleomycin‐induced rat model of pulmonary fibrosis, berberine—traditionally used as an intestinal anti‐infective—demonstrated therapeutic potential by suppressing Smad2/3 signaling, inhibiting the FAK‐PI3K/Akt–mTOR pathway, and restoring autophagy, collectively reversing fibrotic lesions [83]. Experimental evidence indicates that berberine induces autophagy in both AECs and fibroblasts, suggesting a systemic rather than cell type–specific effect. This broad mechanism of action, while beneficial for multitarget modulation, may limit its precision in clinical applications. The subtype‐, cell‐, and stage‐specific nature of macrophage autophagy limits the development of targeted therapies. Nevertheless, several natural compounds have shown therapeutic potential in bleomycin‐induced pulmonary fibrosis. Zukamu Granules restore alveolar macrophage homeostasis by modulating the autophagy–apoptosis balance and suppressing the TLR4/MyD88/NF‐κB pathway [84], while nobiletin activates AMPK and inhibits mTOR to enhance autophagy and suppress M2 polarization [85]. These findings highlight macrophage autophagy–targeted natural compounds as promising strategies for IPF, warranting further exploration of specific targets and delivery approaches. Therapeutic strategies, experimental models, and targets related to autophagy in IPF are summarized in Table 2.

**TABLE 2:** Experimental models and therapeutic strategies targeting autophagy in IPF.

Experimental model	Cell type	Therapeutic approach	Target/Drug	References
Bleomycin‐induced mouse model	ATII cells	Restore autophagy via PLAC8–p53–VCP‐UFD1‐NPLOC4 axis	PLAC8	Sun et al. (2025)
Bleomycin‐induced mouse model	Fibroblasts	Early autophagy induction prevents fibrosis; late treatment ineffective due to ECM stiffening	Rapamycin, Sirolimus	Gui et al. (2015)
General/clinical (IPF)	Fibroblasts	Approved antifibrotic drug; modulates noncanonical autophagy (Beclin1‐dependent, Atg7‐independent)	Nintedanib (multi‐TKI: FGFR, VEGFR, PDGFR)	Rangarajan et al. (2016)
Bleomycin‐induced rat model	—	Restore autophagy and suppress profibrotic signaling	Berberine (inhibits Smad2/3 & FAK–PI3K/Akt–mTOR)	Chitra et al. (2015)
Bleomycin‐induced rat model	Macrophages	Regulates autophagy–apoptosis balance	Zukamu Granules	Li et al. (2022)
Bleomycin‐induced mouse model	Macrophages	Promote autophagy to reduce M2 polarization	Nobiletin	Cheng et al. (2024)

## 4 Necroptosis

Necroptosis is a genetically regulated form of programmed cell death that occurs independently of caspases and relies on interactions among receptor‐interacting protein kinase 1 (RIPK1), RIPK3, and mixed lineage kinase domain‐like protein (MLKL) [86]. It shares upstream signaling with apoptosis, particularly at the level of death receptor activation, where caspase‐8 acts as a critical checkpoint; its inhibition redirects signaling toward RIPK1–RIPK3–MLKL–dependent necroptosis. Following phosphorylation, MLKL oligomerizes and translocates to the plasma membrane, increasing membrane permeability and facilitating the release of intracellular contents. In contrast to apoptosis, necroptosis triggers inflammation through the release of DAMPsand the proinflammatory cytokine IL‐1β [87].

A 2017 study reported elevated necroptosis in IPF lungs, with many AECspositive for RIPK3 or phosphorylated MLKL rather than caspase‐3, indicating a shift toward non‐apoptotic cell death. Necrostatin‐1 treatment or RIPK3 deficiency in mouse primary AECs markedly reduced bleomycin‐induced cell death and fibrosis in mice, providing further evidence for AECs necroptosis [88]. Mechanistically, phosphorylated MLKL oligomerizes and disrupts plasma membranes, releasing DAMPs such as HMGB1 and IL‐1β that amplify inflammation and stimulate fibroblast proliferation and differentiation. Additionally, RIPK3 activates NF‐κB and IL‐1β signaling independently of pore formation, further promoting inflammatory and fibrotic progression [89].

Macrophage necrosis has been observed during the acute exacerbation phase of bleomycin‐ and lipopolysaccharide‐induced pulmonary fibrosis in mice [90]. A similar phenomenon has been reported in silicosis models, where macrophage necrosis correlates positively with inflammation, M1 macrophage polarization, and increased expression of TNF‐α and IL‐6 [91]. However, direct evidence for macrophage necroptosis in human IPF remains limited. In addition, it is unclear whether RIPK3‐associated signaling in macrophages reflects bona fide necroptotic cell death or necroptosis‐independent inflammatory or metabolic functions [92]. Furthermore, the relationship between macrophage necroptosis and macrophage heterogeneity, particularly across distinct macrophage subsets, has not been defined.

Necroptosis in fibroblasts within IPF, however, has been infrequently reported. This may reflect the reduced surface expression of Fas on IPF fibroblasts, which limits the formation of downstream necroptosis‐associated complexes. High levels of DcR3 and NFATc1, which sequester FasL, further suppress Fas signaling. Notably, Lee and colleagues observed the coexistence of apoptosis and necroptosis in AEC2s in a mouse model, with overlap at the signaling pathway level. Consequently, it is plausible that the apoptosis‐resistant phenotype of IPF fibroblasts also confers resistance to necroptosis [88].

## 5 Ferroptosis

Ferroptosis is an iron‐dependent form of RCD characterized by lipid peroxidation and glutathione depletion [93]. It arises from cellular stress conditions—particularly oxidative imbalance and metabolic disruption—that are also engaged by other death pathways. Under conditions of impaired redox homeostasis and iron overload, these stress signals preferentially drive cells toward ferroptotic death. It displays distinct features that differentiate it from other forms of RCD, including apoptosis, necroptosis, and pyroptosis [94]. Cellular dysfunction and death arise from an imbalance between ROS generation and detoxification, resulting in lipid peroxidation, protein modifications, and DNA damage. Significant iron accumulation has been observed in lung tissue from patients with IPF and in experimental models of bleomycin‐induced pulmonary fibrosis, correlating with disease severity and declining lung function [95, 96]. Furthermore, bioinformatic analyses have revealed a strong association between ferroptosis‐related genes and IPF development. Certain ferroptosis‐associated genes detected in BALF also exhibit prognostic value in IPF patients [97, 98, 99].

### 5.1 Ferroptosis in Macrophage

Compared with healthy controls, AMsfrom patients with IPF show marked iron accumulation [100, 101]. Rather than a passive bystander, macrophage iron accumulation may link iron dysregulation to oxidative stress and profibrotic signaling in IPF. Evidence from knockout studies indicates that this accumulation contributes to fibrogenesis and lung function decline. The transferrin receptor CD71 (TfR1) mediates transferrin‐bound iron uptake in AMs, and the proportion of CD71^+^ AMs increases significantly in bleomycin‐induced models, an effect mitigated by deferoxamine (DFO) [102]. A 2016 clinical study further associated high CD71 expression with poorer survival in IPF. The mechanisms underlying macrophage iron overload remain uncertain, potentially involving CD71 upregulation, erythrocyte engulfment, or other pathways. Iron accumulation enhances ROS production, which not only promotes oxidative injury but also drives functional reprogramming of macrophages. Iron and iron‐dependent ROS levels were found to be markedly elevated in BALF and AMs from IPF patients, and were correlated with allelic variations in the HFE gene [103]. Importantly, iron overload per se does not necessarily equate to ferroptosis, but may first induce a sublethal state characterized by oxidative stress and inflammatory activation. In addition, silica‐induced ferroptosis in macrophages promotes profibrotic cytokine release and accelerates fibrosis progression [104].

The impact of iron overload on macrophage polarization remains controversial. Some studies indicate that iron overload enhances the expression of M1 markers, including IL‐6, TNF‐α, and IL‐1β, while reducing M2 markers such as TGM2 [105], whereas other evidence indicates that iron promotes M2 polarization in THP‐1–derived macrophages [106]. These seemingly conflicting observations may suggest that iron overload does not drive a fixed polarization state, but rather induces a dysregulated macrophage phenotype with both inflammatory and profibrotic features. In IPF, M1 polarization is generally considered antifibrotic and M2 polarization pro‐fibrotic, though excessive M1 activity may also worsen fibrosis. Overall, fibrosis appears to result not from a single polarization state but from an imbalance between M1 and M2 phenotypes. Both iron overload and ferroptosis in macrophages accelerate IPF progression by disrupting macrophage function and stimulating profibrotic cytokine release that amplifies tissue fibrosis. However, it remains unclear whether macrophage ferroptosis represents a primary driver of fibrosis or a secondary consequence of persistent iron overload and oxidative stress. Moreover, most current evidence relies on indirect markers, and whether ferroptosis occurs in specific macrophage subsets in vivo, and how it temporally relates to other cell death programs remains to be determined.

### 5.2 Ferroptosis in Epithelial Cells

Iron accumulation and ferroptosis in AECs were first demonstrated in a bleomycin‐induced mouse model of pulmonary fibrosis, where treatment with the ferroptosis inhibitor DFOreduced iron overload and markedly attenuated fibrosis [95]. In bleomycin‐ and SiO_2_‐induced models, AEC2s exhibited ferroptosis with downregulation of GPX4 and FSP1 mediated by UHRF1, which recruited DNMT1 to their promoters to enhance DNA methylation and suppress expression [107]. Loss of GPX4, a selenium‐dependent enzyme that eliminates lipid peroxides, leads to membrane damage and ferroptotic death [108, 109, 110]. Moreover, ferroptosis in type II AECs is closely linked to aging. In a bleomycin‐treated mouse model, ferroptosis‐related genes in AEC2s of both young and aged mice showed altered expression within 4 days of treatment; while only AEC2s from young mice demonstrated recovery by Day 14, indicating greater susceptibility in aging cells [111]. Given that cellular aging is integral to IPF pathogenesis, its interaction with ferroptosis likely amplifies epithelial injury and fibrotic progression. Despite these advances, several questions remain. It is unclear whether AEC2 ferroptosis is an early event or a late consequence of persistent injury. Current evidence largely relies on indirect markers such as GPX4 and lipid peroxidation, with limited in vivo validation. The mechanisms underlying increased susceptibility in aged AEC2s, and whether ferroptosis occurs in specific cell subsets or in coordination with other death programs, also remain to be clarified.

### 5.3 Ferroptosis in Fibroblasts

In fibroblasts, iron accumulation promotes proliferation and activation rather than cell death, consistent with the concept that iron overload accelerates fibrotic progression in lung tissue. Exposure of human lung fibroblasts to ferric ammonium citrate (FAC) (10 or 50 μM) increased proliferation and upregulated IL‐6, IL‐8, while 10 μM FAC also increased mRNA levels of ECM‐related genes, including COL1A2 and TNC [102]. Subsequent research in 2022 further showed that TGF‐β stimulation elevates transferrin receptor (TFRC) expression via the TAZ–TEAD4 complex, enhancing intracellular Fe^2+^ and driving fibroblast‐to‐myofibroblast transition marked by α‐SMA and COL1A1 upregulation without inducing ferroptosis [112]. Transcriptomic analyses revealed activation of lipid and cholesterol metabolism and PPAR signaling genes (SCD, FADS2, ACSL3); these genes have been proven to promote the migration and invasion of cancer cells and are associated with poor prognosis [113, 114, 115, 116]. Such metabolic adaptations may influence membrane composition and reduce susceptibility to lipid peroxidation, thereby potentially limiting ferroptotic sensitivity, although direct evidence in fibroblasts remains limited. Concurrent induction of SLC40A1 and ferritin heavy chain further limited free iron, supporting sustained fibroblast activation and fibrotic progression.

Ferroptosis in fibroblasts may be amplified through crosstalk with other ferroptotic cells. Macrophages release pro‐inflammatory and profibrotic cytokines such as TNF‐α and IL‐1β during ferroptosis [104], while ferroptotic AEC2s secrete mediators that similarly enhance fibrogenesis [117]. In this context, fibroblasts that are relatively resistant to ferroptosis may instead respond to these signals by undergoing activation and myofibroblast differentiation, thereby promoting excessive ECM deposition and reinforcing fibrosis.

### 5.4 Ferroptosis in Endothelial Cells

Although direct experimental validation IPF remains unavailable, endothelial ferroptosis has been hypothesized to contribute to vascular injury and fibrotic progression. Early IPF lesions often show capillary rarefaction and iron deposition, implying potential disturbances in iron metabolism and oxidative stress. Supportive evidence arises from non‐IPF models. In pulmonary arterial hypertension, ECs exhibit ferroptotic signatures (GPX4↓, ACSL4↑, Fe^2+^ accumulation), and ferroptosis inhibition alleviates vascular remodeling and inflammation [118]. Enrichment of ferroptosis‐related genes in endothelial subsets has also been observed, suggesting links to chronic endothelial inflammation [119]. Findings from different experimental settings implicate Calpain‐1 as a regulator of endothelial ferroptosis. In a TGF‐β–stimulated endothelial model, Calpain‐1 enhanced TFRC expression and iron uptake [120]; in a hypoxia‐induced injury model, it aggravated mitochondrial ROS generation, reversed by ginsenoside Rg1 [121]; and in a vascular remodeling model, it promoted ferroptosis via the AMPK–YAP–ACSL4/TfR1 axis [122]. While these studies originate from non‐IPF contexts, they highlight Calpain‐1–mediated oxidative stress and iron dysregulation as possible contributors to endothelial injury under the hypoxic and TGF‐β–rich milieu characteristic of IPF.

### 5.5 Potential Therapeutic Strategies Related to Ferroptosis

Current therapeutic strategies targeting ferroptosis in IPF focus on regulating iron homeostasis, inhibiting lipid peroxidation, and enhancing antioxidant systems. Iron chelators, such as DFO and CQ, reduce intracellular free iron and suppress Fenton reaction–mediated ROS production, significantly attenuating fibrosis in bleomycin‐induced models [96, 123]. Inhibition of ACSL4 by rosiglitazone activates PPAR‐γ, upregulating PTEN and reducing TGF‐β levels, providing protection in paraquat‐induced rat models [124]. Radical‐trapping antioxidants, like Ferrostatin‐1, scavenge lipid peroxides and exhibit antifibrotic efficacy in silicosis models [104]. Selenomethionine and ebselen protect lung cells by suppressing inflammatory pathways such as cGAS/STING/NF‐κB and MAPK‐TGF‐β, respectively, and have shown promise in animal models of fibrosis [125, 126]. Although preclinical evidence supports ferroptosis inhibition as a promising antifibrotic strategy, translation to clinical practice will require validation of specificity, long‐term efficacy, and safe delivery to the lung. Table 3 compiles the ferroptosis‐related treatment strategies, experimental models, and therapeutic targets discussed above.

**TABLE 3:** Experimental models and therapeutic strategies targeting ferroptosis in IPF.

Experimental model	Cell type	Therapeutic approach	Target/Drug	References
Bleomycin‐induced mouse model	Alveolar epithelial A549 cells	Inhibit iron overload, block ROS via Fenton reaction suppression	Deferoxamine (DFO)	Takahashi et al. (2021)
Bleomycin‐induced mouse model	Fibroblasts	Inhibit iron overload, block ROS via Fenton reaction suppression	Chloroquine (CQ)	Zhu et al. (2021)
Paraquat‐induced rat model	—	Activate PPAR‐γ, increase PTEN expression and decrease TGF‐β	Rosiglitazone (ACSL4 inhibitor)	Zhang et al. (2019)
Silicosis‐induced mouse model	Macrophages	Inhibit lipid peroxidation and cell death, regulate ferroptosis‐related genes expression	Ferrostatin‐1 (Fer‐1)	Liu et al. (2022)
Bleomycin‐induced mouse model	—	Suppresse the increase in oxidized diacylglycerol (DAG)	Ebselen	Yagasaki et al. (2022)
PM2.5‐induced mouse model	AEC2s	Inhibite PM2.5‐induced inflammatory response and cellular senescence by hindering cGAS/STING/NF‐κB pathway	Selenomethionine (Se‐Met)	Wang et al. (2021)

## 6 Pyroptosis

Pyroptosis was first identified in macrophages undergoing caspase‐1–dependent death during bacterial infection [127]. It is now recognized as a distinct, gasdermin (GSDM)‐mediated form of regulated necrosis, characterized by cellular swelling, lysis, and release of pro‐inflammatory mediators [128, 129]. Activation of inflammasomes—complexes comprising pattern recognition receptors and caspase‐1—initiates this process. Upon activation, caspase‐1 cleaves gasdermin D (GSDMD), liberating its N‐terminal fragment, which inserts into the plasma membrane to form pores and induce cell rupture [130, 131, 132, 133, 134, 135, 136]. Concurrently, caspase‐1 converts pro‐IL‐1β and pro‐IL‐18 into their active forms, amplifying inflammation. Together, these coordinated molecular events define pyroptotic cell death and its potent inflammatory consequences.

Pyroptosis is increasingly recognized as a key immune defense mechanism and a driver of lung inflammation and fibrosis. In pulmonary injuries caused by silica, asbestos, mechanical stress, IPF, and cystic fibrosis, inflammasome activation correlates with elevated pro‐inflammatory cytokines and fibrotic responses [137, 138]. In an LPS‐induced mouse model, increased caspase‐1 and IL‐1β levels in BALF indicate strong inflammasome‐mediated pyroptosis during acute lung injury and subsequent fibrosis [139]. Pyroptosis‐related genes (PRGs) are differentially expressed between healthy and IPF lungs, and an eight‐gene PRG signature has shown strong prognostic value for IPF progression [140].

**FIGURE 3:**
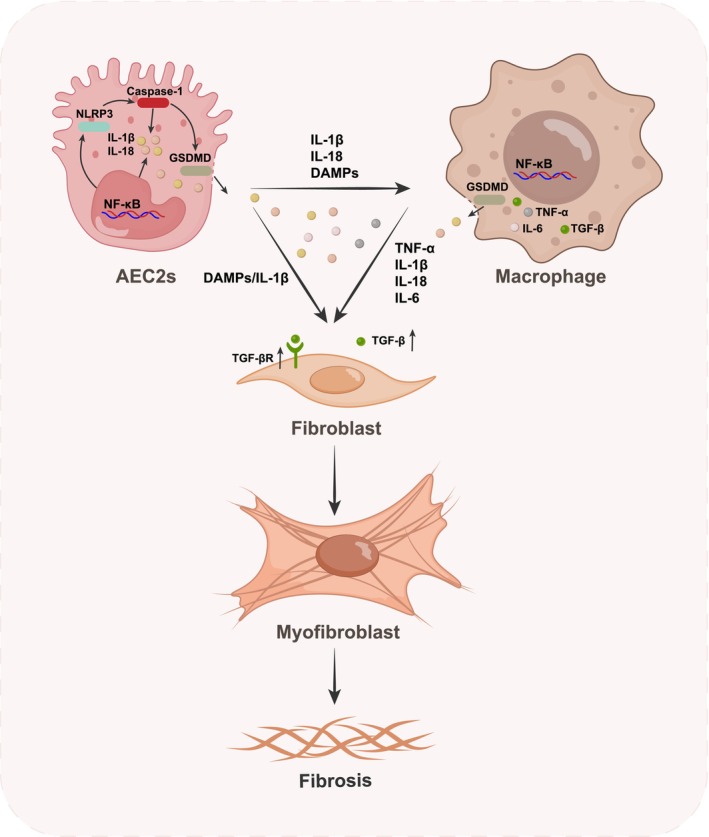
Schematic illustration of pyroptosis‐driven inflammation and fibrogenic signaling in IPF. Injury to AEC2s initiates the priming and activation of the NLRP3 inflammasome, leading to caspase‐1–mediated cleavage of GSDMD and formation of membrane pores that trigger AEC2s pyroptosis. The release of IL‐1β, IL‐18, and damage‐associated molecular patterns (DAMPs) recruits and activates macrophages, which undergo inflammasome assembly and pyroptosis in response to DAMPs and pathogen‐associated molecular patterns (PAMPs). Activated macrophages secrete inflammatory and profibrotic mediators, including TNF‐α, IL‐6, and TGF‐β, amplifying the inflammatory milieu and promoting fibroblast activation. Exposure of fibroblasts to these cytokines, together with DAMPs released from injured AEC2s, upregulates TGF‐β receptor expression and drives their differentiation into α‐smooth muscle actin–positive myofibroblasts. These myofibroblasts deposit extracellular matrix components, such as fibronectin and type I collagen, culminating in progressive pulmonary fibrosis.

In IPF, pyroptosis originates from AEC injury and amplifies a macrophage pyroptosis–inflammation loop, culminating in fibroblast‐to‐myofibroblast differentiation [141], as summarized in Figure 3. These interconnected processes constitute a unified pathological axis and should be considered as a single mechanistic continuum. Mitochondrial DNA leakage, oxidative stress, and endoplasmic reticulum stress constitute core stress axes that intersect with multiple RCD pathways. In the presence of inflammasome‐activating cues, including TLR4/NF‐κB–mediated priming and triggers such as K^+^ efflux, ROS burst, and lysosomal rupture, these signals are routed through NLRP3 inflammasome activation as a key checkpoint, leading to caspase‐1–mediated GSDMD cleavage and AEC pyroptosis [141, 142, 143]. The subsequent release of DAMPs, IL‐1β, and IL‐18 recruits macrophages, monocytes, and lymphocytes, propagating inflammation. In macrophages, DAMPs and PAMPs engage PRRs, activating caspase‐1 to cleave GSDMD and mature IL‐1β/IL‐18, which are released through GSDMD pores. These molecular events not only induce macrophage pyroptosis but also propagate an inflammatory signaling cascade across the lung, reinforcing tissue injury and fibrotic progression. Excessive activation of M1 macrophages, accompanied by a phenotypic shift toward M2 polarization, represents another downstream consequence, further promoting AEC pyroptosis and the secretion of pro‐inflammatory cytokines along with TGF‐β [141]. Endothelial pyroptosis may also occur in IPF, but direct evidence from IPF or fibrotic models is lacking, with current findings derived mainly from studies on pulmonary ischemia–reperfusion or acute lung injury [144, 145, 146, 147, 148]. Under sustained cytokine and TGF‐β stimulation, fibroblasts differentiate into myofibroblasts that deposit fibronectin and collagen I, driving fibrosis [149, 150]. In macrophages, AIM2 and NLRP3 inflammasome dysregulation promotes fibrotic remodeling, while GSDMD knockdown suppresses silica‐induced pyroptosis and fibroblast activation [151, 152, 153]. Collectively, these findings identify inflammasome activation and GSDMD‐mediated pyroptosis as central drivers linking epithelial injury, inflammation, and fibrotic progression in IPF.

Recent advances in understanding pyroptosis have revealed multiple therapeutic targets for IPF, including inflammasome inhibition, caspase‐1 blockade, GSDMD interference, IL‐1β neutralization, and modulation of upstream signaling. Among these, the NLRP3 inflammasome is a key intervention point. Small‐molecule inhibitors such as lycorine, iguratimod, elamipretide, and betanin suppress NLRP3 activation and downstream pyroptosis, reducing inflammation and fibrotic remodeling [154, 155, 156, 157]. The approved antifibrotic drug pirfenidone similarly limits ROS‐dependent NLRP3 activation and decreases IL‐1β and TGF‐β1 production [139]. Caspase‐1 inhibitors like VX‐765 mitigate silica‐ and bleomycin‐induced fibrosis by directly suppressing caspase‐1 activity [158]. At the effector level, GSDMD inhibition with disulfiram—an FDA‐approved drug for alcohol use disorder—blocks pore formation and reduces fibrosis in systemic sclerosis and pulmonary models [159, 160]. Activation of the COX‐2/PGE2 pathway further links disulfiram's antifibrotic effect to suppression of NLRP3 and caspase‐1 activity [161, 162, 163]. Upstream regulation also offers therapeutic potential: Liproxstatin‐1, a lipid peroxidation inhibitor, reduces fibrosis via the ROS/p53/α‐SMA axis [164], while P2X7R antagonists such as A438079 disrupt the P2X7/NLRP3 pathway and suppress fibrotic remodeling [165]. These findings support pyroptosis inhibition—spanning upstream triggers to terminal executioners—as a promising antifibrotic strategy warranting clinical evaluation and combinatorial optimization. Future research should prioritize clinical evaluation of these interventions and investigate combination approaches to maximize efficacy in IPF. An overview of pyroptosis‐targeted therapeutic interventions, experimental models, and molecular targets in IPF is presented in Table 4.

**TABLE 4:** Experimental models and therapeutic strategies targeting pyroptosis in IPF.

Experimental model	Cell type	Therapeutic approach	Target/Drug	References
Bleomycin‐induced mouse model	Macrophages	NLRP3 inhibitors	Lycorine	Liang et al. (2020)
Bleomycin‐induced mouse model	Macrophages	Regulates macrophage polarization through the TLR4/NF‐κB pathway	Iguratimod	Xu et al. (2025)
Bleomycin‐induced mouse model	Macrophages	Inhibits the activation of NLRP3 inflammasome by restoring/protecting mitochondrial function	Elamipretide(SS‐31)	Nie et al. (2023)
Bleomycin‐induced rat model	—	Inhibits the NLRP3 inflammasome cascade reaction	Betanin	Abd Elrazik et al. (2024)
LPS‐induced ALI and fibrosis(mouse model)	Macrophages	Inhibits ROS‐mediated activation of the NLRP3 inflammasome	Pirfenidone	Li et al. (2018)
Silica‐induced mouse model	Macrophages	Caspase‐1 blockade	VX‐765	Tao et al. (2023)
Bleomycin‐induced mouse model	Primary DHLF‐IPF cells; A549 cells	Upregulates COX‐2/PGE2 pathway (potentially alleviates pyroptosis)	Disulfiram	Pei et al. (2025)
Bleomycin‐induced mouse model	A549 cells	ROS modulation	Liproxstatin‐1	Tao et al. (2021)

## 7 Curoptosis

Cuproptosis is a copper‐dependent form of RCD in which copper ions disrupt cellular metabolism by binding to acetylated proteins of the tricarboxylic acid (TCA) cycle [166]. This interaction triggers protein aggregation, loss of iron–sulfur cluster proteins, and oxidative stress, culminating in proteotoxicity and cell death [167]. Mitochondrial metabolism is central to this process, as cells dependent on oxidative phosphorylation are particularly susceptible to copper‐induced toxicity [168]. While this places cuproptosis within the mitochondrial regulatory axis shared with apoptosis and ferroptosis, copper binding to lipoylated TCA cycle proteins diverts this stress toward a distinct proteotoxic form of cell death.

Although cuproptosis has recently emerged as a novel form of RCD, its involvement in IPF remains poorly defined. Most current studies in lung tissues describe copper dysregulation and copper‐induced cellular stress or toxicity, whereas direct evidence for canonical cuproptosis is still lacking. As early as the 1990s, epidemiologic and experimental evidence suggested that environmental copper exposure may be a risk factor for pulmonary fibrosis [169, 170]. Inhaled copper particles accumulate in lung tissue, inducing oxidative stress, inflammation, and EMT, which activate fibroblasts and promote collagen deposition [171]. Subsequent studies showed that copper oxide nanoparticles trigger ROS production, upregulate p38 and p53 signaling, and cause DNA damage [172]. Additionally, copper oxide enhances mitochondrial ROS generation, activates MAPKs, and induces MMP‐3 expression. These molecular changes drive EMT, marked by increased vimentin, α‐SMA, and fibronectin, facilitating pulmonary fibrosis progression [173]. More recently, multiple cuproptosis‐related genes—including CFH, STEAP1, HDC, NUDT16, and FMO5—were identified through bioinformatic analysis as differentially expressed in IPF and possessing potential diagnostic value [174, 175]. Future studies should aim to delineate the complete canonical cuproptosis pathway in IPF models, thereby clarifying whether cuproptosis represents a distinct pathogenic mechanism or part of broader copper‐induced stress responses.

### 7.1 Cuproptosis in Epithelial Cells

Lung epithelial cells, particularly AEC2s, are pivotal in the initiation of IPF, as recurrent microinjury compromises repair capacity and promotes fibrotic remodeling. Cuproptosis appears to exacerbate this process through several mechanisms, some of which may involve cuproptosis‐related pathways. Copper exposure, including CuSO_4_ or CuO nanoparticles, induces EMT, primarily driven by oxidative stress, ROS generation, and activation of MAPK signaling pathways independent of Smad signaling [176]. While cuproptosis‐related genes such as FDX1 may act to suppress EMT progression, excessive copper disrupts this regulatory balance and enhances fibrotic remodeling. For instance, copper‐induced mitophagy in A549 cells triggers EMT, which can be effectively prevented by melatonin or by autophagy inhibitors [9]. Moreover, copper‐induced oxidative stress promotes apoptosis in epithelial cells via copper‐catalyzed ROS production through the Fenton reaction, leading to DNA damage, p53 activation, and mitochondrial membrane depolarization. These alterations result in the release of cytochrome c and AIFM1, thereby activating both caspase‐dependent and caspase‐independent apoptotic pathways [177]. Concurrently, combined exposure of lung epithelial cells to ethyl maltol and copper markedly elevates ROS levels and upregulates ferritin light chain and HO‐1 expression, ultimately promoting apoptosis [178]. Copper overload further reduces the activity of antioxidant enzymes, including GSH‐Px and SOD, while increasing lipid peroxidation, thereby sensitizing cells to oxidative injury [179]. In addition, smoking‐related upregulation of cuproptosis‐associated genes, such as DLD and CDKN2A (p16), in airway epithelia links copper toxicity to apoptosis and aging‐like phenotypes [180].

Bronchial epithelial cells are likewise vulnerable to copper‐induced cell death. In vitro, combined exposure to CuCl_2_ and elesclomol markedly induces death of BEAS‐2B cells, an effect that cannot be prevented by inhibitors of apoptosis or necrosis. Excess copper also promotes EMT in these cells. Notably, both copper‐induced cell death and EMT can be reversed by the copper chelator tetrathiomolybdate (TTM). In vivo, TTM demonstrates significant therapeutic efficacy in bleomycin‐induced pulmonary fibrosis in mice, underscoring the regulation of copper metabolism as a novel and promising strategy for mitigating fibrotic progression [181].

Despite these findings, several questions remain. It is unclear whether epithelial cell death in this context represents true cuproptosis or more general copper‐induced cytotoxicity, as current evidence does not clearly distinguish between these processes. Direct evidence for canonical cuproptosis—such as FDX1‐dependent mitochondrial protein lipoylation and TCA cycle disruption—in primary AEC2s is still lacking. Current evidence in epithelial cells may therefore reflect a broader spectrum of copper‐induced stress responses, rather than a clearly defined cuproptosis pathway. In addition, the relationship between copper‐induced mitochondrial changes, apoptosis, and EMT remains poorly defined, and most existing data are derived from transformed cell lines or acute exposure models, which may not reflect the chronic conditions of IPF. Future studies should therefore focus on validating cuproptosis in primary AEC2s in vivo, clarifying its distinction from other forms of copper‐induced cell death, and determining whether targeting copper metabolism can be applied in a cell‐specific and clinically safe manner.

### 7.2 Cuproptosis in Fibroblasts

Copper metabolism appears to influence fibroblast behavior and fibrotic remodeling. In IPF, copper dyshomeostasis may regulate fibroblast activation through both cell‐intrinsic and microenvironmental mechanisms. Diminished cuproptosis allows persistent fibroblast activation, contributing to fibrotic foci formation. As fibroblasts differentiate into myofibroblasts, cuproptosis‐related genes like FDX1, LIAS, DLD, PDHA1, PDHB, DLAT, and LIPT1 are downregulated, as shown by single‐cell RNA sequencing of IPF models and patient samples [175]. This downregulation is associated with a profibrotic phenotype characterized by ECM deposition and elevated expression of markers such as α‐SMA. These findings suggest that fibroblasts acquire reduced cuproptosis susceptibility during myofibroblast transition, resembling the apoptosis resistance that sustains fibroblast accumulation in fibrotic foci. However, this does not imply that lower copper is uniformly profibrotic. Rather, copper homeostasis, rather than absolute copper abundance, appears to be critical. Copper supplementation in other tissues reduces myofibroblast proportions by inhibiting TGF‐β1 secretion, suggesting a protective role in the lung when copper is balanced [182]. At the same time, the consequences of copper exposure likely depend on intercellular interactions within the fibrotic niche. While copper may promote fibroblast activation through paracrine signals from epithelial cells or macrophages, copper deficiency or TTM chelation inhibits this process by reducing lysyl oxidase activity and collagen cross‐linking [173]. Consistently, in bleomycin‐induced pulmonary fibrosis, TTM attenuated fibrosis while lowering lung copper levels, reducing Olig‐DLAT accumulation, and suppressing EMT/FMT‐associated profibrotic markers, including α‐SMA, collagen deposition, and TGF‐β signaling [181]. Together, these findings support a model in which fibroblasts in IPF become less sensitive to cuproptosis, whereas copper imbalance in the fibrotic lung microenvironment further amplifies fibrogenesis.

A key unresolved issue is how increased tissue copper burden coexists with reduced cuproptosis susceptibility in fibroblasts. This may reflect cell‐type‐specific regulation, whereby copper overload promotes epithelial injury and profibrotic remodeling, while fibroblasts simultaneously escape cuproptosis through repression of cuproptosis‐related genes. Future studies should therefore use cell‐type‐specific and spatially resolved approaches to define copper distribution, labile copper pools, and cuproptosis sensitivity across fibroblasts, epithelial cells, and macrophages, and to determine whether copper modulation acts directly on fibroblast fate or mainly through remodeling of the fibrotic microenvironment.

### 7.3 Cuproptosis in Macrophages

Macrophages employ copper transporters such as CTR1 and ATP7A to maintain antimicrobial defense, yet copper imbalance can lead to toxicity. In IPF, a more coherent view is that copper‐related pathways may promote macrophage‐driven fibrosis by affecting both macrophage function and macrophage fate. Environmental copper exposure may impair macrophage function, promoting inflammation and fibrosis through abnormal cytokine release. More specifically, cuproptosis‐related pathways appear to influence macrophage persistence and polarization within the fibrotic niche. The cuproptosis‐related gene CDKN2A is upregulated in IPF lung tissue, particularly in profibrotic M2 macrophages, where it may confer resistance to cuproptosis, promoting their persistence and enhancing fibrosis. Inhibiting CDKN2A with palbociclib sensitizes M2 macrophages to cuproptosis and reduces their abundance [183]. Additional pharmacological evidence suggests that copper‐related agents can also modulate macrophage behavior; for example, disulfiram inhibits monocyte and macrophage migration by interfering with CCR2 and CCR5 signaling [184]. Together, these findings support a model in which copper dysregulation contributes to IPF not only by disturbing macrophage inflammatory responses but also by sustaining profibrotic macrophage states through reduced cuproptosis sensitivity. However, current evidence remains largely correlative and pharmacological, and it is still unclear whether macrophage cuproptosis itself directly drives profibrotic macrophage persistence and fibrosis progression. Moreover, the use of the M1/M2 framework may oversimplify macrophage heterogeneity in IPF, and the apparent coexistence of copper toxicity and cuproptosis resistance will require clarification at the level of specific macrophage subsets, disease stages, and intracellular copper pools.

## 8 Crosstalk and Integrated Regulation Among Different Cell Death Pathways in IPF

### 8.1 Conceptual Framework for RCD Crosstalk in IPF

IPF lungs are chronically subjected to a sustained, multifaceted stress milieu—including oxidative and ER stress, mitochondrial dysfunction, metabolic imbalance, and persistent inflammatory signaling—across alveolar epithelial, fibroblast, and immune compartments. These stress states are widely viewed as core drivers of maladaptive repair and progressive fibrotic remodeling. Consistent with this, multiple RCD programs have been implicated in IPF: epithelial apoptosis is a hallmark lesion, and emerging evidence supports RIPK3‐dependent necroptosis in fibrotic lung injury [88]. In addition, UPR markers can be upregulated and locally co‐localize with apoptosis‐ and autophagy‐associated molecules in IPF epithelium, suggesting that multiple death‐related modules may coexist within the injured epithelial niche [66].

Mechanistically, IPF‐relevant stressors are unlikely to feed into single, insulated death programs. ER stress has been linked to epithelial apoptosis [185], while oxidative stress/ROS and mitochondrial dysfunction promote apoptosis [186] and intersect with pathways implicated in ferroptosis [187] and pyroptosis [188]. Together, these data suggest that shared stress inputs can tune multiple RCD modules, and that similar RCD outcomes may emerge from distinct stress constellations. While prior IPF studies have elucidated individual RCD modalities and some common regulators, a key gap is how these programs are coordinated under chronic multi‐stress—whether co‐engaged within the same cells, partitioned into stage‐dominant states, or connected via context‐dependent switching. This motivates treating RCD as a dynamic network rather than strictly discrete modules.

This integrated view may be especially pertinent in IPF, where recurrent microinjury, aberrant repair, and failed restoration of homeostasis create a chronic stress landscape that could favor “multi‐stressor–multi‐death” responses. Across models of infection, cancer, and inflammatory disease, stress signaling networks are increasingly recognized to coordinate multiple RCD pathways via shared regulatory nodes [189]. The integrated stress response (ISR) and stress granules (SGs) have been proposed in other contexts as candidate organizing mechanisms that co‐regulate death‐related factors under stress, potentially tuning activation thresholds across apoptosis, inflammasome/pyroptosis, and necroptosis. While ER stress/UPR signaling is well studied in IPF and partially overlaps with the ISR [190], whether analogous integrative mechanisms operate in fibrotic lungs to coordinate RCD engagement and pathway choice remains unclear; direct evidence is currently limited, representing a key knowledge gap.

Several shared regulatory nodes provide plausible substrates for crosstalk and convergence among RCD pathways. The caspase‐8–RIPK1 platform is a canonical gatekeeper for apoptosis–necroptosis bifurcation [191]. Mitochondrial dysfunction can drive apoptosis [186] and favor ferroptosis via ROS amplification and lipid peroxidation [187]; while sustained ROS can promote inflammasome activation [188] and amplify inflammatory death signaling. Collectively, these nodes may act as integrative hubs that, depending on stress intensity, metabolic state, and inflammatory context, function as “selection platforms” biasing cells toward specific death execution programs.

**FIGURE 4:**
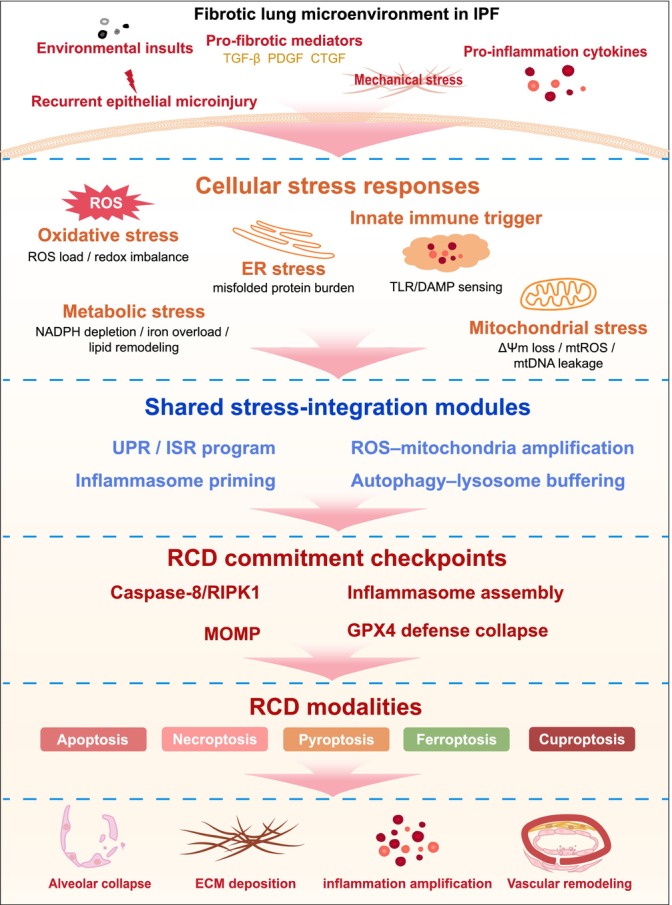
A multi‐stress–integration–checkpoint framework linking RCD to IPF pathology. This schematic provides a hypothesis‐generating conceptual framework for how IPF, as a chronic multi‐stress condition, may coordinate multiple RCD programs within the same cell. Instead of operating as isolated pathways, RCD modalities may converge on shared stress inputs and integrative hubs, with context‐dependent gating/commitment checkpoints determining the dominant execution program. This framework emphasizes the interconnected nature of the stressors, pathways, and decision points within the cellular environment. IPF microenvironment: IPF lesions develop in a persistently disturbed extracellular niche shaped by environmental insults and recurrent epithelial microinjury. ECM stiffening imposes chronic mechanical cues, while inflammatory cytokines and pro‐fibrotic mediators further condition the local milieu. Cellular stress responses: Within exposed or injured cells, this niche is mirrored by overlapping stress states, including oxidative/redox stress, ER stress, metabolic stress, innate/inflammatory activation, and mitochondrial stress/dysfunction. Shared stress‐integration modules: Stress inputs converge on shared modules—UPR/ISR, ROS–mitochondria amplification, inflammasome priming, and autophagy–lysosome buffering. Autophagy modulates stress burden and sets the context for checkpoint engagement; when buffering is insufficient, stress may accumulate, lowering the threshold for regulated cell death. RCD commitment checkpoints: Commitment checkpoints (caspase‐8–RIPK1, MOMP, inflammasome–caspase–gasdermin activation, and the lipid peroxidation threshold/GPX4 defense collapse) are depicted as gating points where integrated signals are filtered into dominant execution outcomes, with checkpoint behavior tuned by cellular context and local thresholds. RCD modalities: Downstream, cells may express a dominant RCD phenotype—apoptosis, necroptosis, pyroptosis, ferroptosis, and where supported by emerging evidence, cuproptosis—used here as organizing categories rather than mutually exclusive states. Pathological outputs: At the tissue level, these RCD programs contribute to key IPF outcomes, including AEC2 epithelial loss, alveolar collapse, inflammation amplification, ECM deposition, and vascular endothelial remodeling, all of which reinforce maladaptive repair and drive fibrotic progression.

Building on evidence for multiple RCD modalities in IPF and their points of intersection, we propose a speculative conceptual framework in which IPF‐associated RCD pathways should not be viewed as isolated, parallel events. Rather, RCD programs may form a dynamic death network shaped by shared stresses, common regulatory nodes, and context‐dependent fate selection (Figure 4). In this view, chronic stress can “prime” multiple RCD modules within the same cell, whereas commitment to a dominant execution program is set by local signal integration within the microenvironment and further modulated by cellular state, spatial lesion heterogeneity, and cell‐type–specific stress sensitivity. For example, under TNF‐α, intact caspase‐8 favors apoptosis, while caspase‐8 inhibition redirects signaling toward necroptosis; ROS can engage apoptosis or, via GSDME, shift execution toward pyroptosis‐like outcomes. Consistently, injured AEC2s are often apoptosis‐prone, whereas fibroblasts in fibrotic niches can be comparatively resistant to apoptotic cues—illustrating how similar stress landscapes can produce divergent outcomes across cells and contexts.

As a hypothesis‐generating framework, this model highlights several priorities for future work: resolving whether multiple RCD programs are co‐engaged within single cells or segregated into stage‐dominant states across lesions; defining how shared stress‐integration modules tune threshold behavior at commitment checkpoints to enable sequential handoffs or compensatory switching; and identifying which shared hubs/checkpoints exert the strongest causal control over injury amplification and maladaptive repair. Therapeutically, if RCD programs in IPF are coupled through shared stress circuitry and gatekeeping platforms, single‐pathway inhibition may be insufficient; instead, targeting upstream stress networks, key integrative hubs, and/or rational combinations may more effectively limit epithelial loss, dampen inflammatory amplification, and constrain maladaptive repair.

Within this framework, direct molecular links between RCD pathways can be viewed as specific implementations of a broader shared stress–integrative hubs–death selection logic. The following sections therefore synthesize evidence for pairwise crosstalk among major RCD modalities and consider the mechanistic and pathological implications of these interactions in IPF.

### 8.2 Autophagy and Apoptosis

In IPF, the crosstalk between autophagy and apoptosis represents an important component of the broader stress‐response network that shapes cell fate and disease progression. Autophagy predominantly serves a cytoprotective role, modulating apoptosis via overlapping signaling pathways, including Beclin‐1, Bcl‐2, and mTOR. At the cellular level, autophagy facilitates the clearance of damaged organelles and misfolded proteins, thereby reducing cellular stress and restraining apoptosis. Conversely, when autophagic activity is compromised, cells may exhibit either increased or decreased susceptibility to apoptosis, contingent on the specific cell type. This imbalance contributes to AEC injury and the persistence of fibroblasts [65]. This balance between autophagy and apoptosis may function as a critical determinant of cell death susceptibility, setting the threshold at which stressed cells commit to apoptosis or maintain survival.

In AECs, impaired autophagy, frequently linked to aging or hyperactivation of mTORC1, results in protein misfolding and increased oxidative stress. These disturbances intensify UPR signaling and trigger apoptosis, which subsequently drives EMT and fibrosis progression [62, 72, 81]. In contrast, stimulation of autophagy, for instance through PLAC8 overexpression, promotes the degradation of pro‐apoptotic proteins including p53, diminishes caspase‐3 activation, and enhances epithelial cell survival [81]. These observations further suggest that shared regulatory nodes linking autophagy and apoptosis may drive distinct cell fate outcomes in a cell type‐dependent manner in IPF.

In fibroblasts and myofibroblasts, autophagy deficiency exerts an opposite effect. It maintains mTORC1 activity and upregulates anti‐apoptotic proteins, including Bcl‐2, thereby attenuating apoptosis while promoting ECM production and cell proliferation. This mechanism reinforces fibrotic remodeling and contributes to the persistence of tissue scarring [29, 66, 192].

Across diverse cell types, the interplay between autophagy and apoptosis forms a dynamic regulatory network. Dysregulation within one cell population can affect others through paracrine signaling, thereby amplifying fibrotic responses. For example, apoptotic AECs release TGF‐β, which suppresses autophagy in fibroblasts, enhances their resistance to apoptosis, and promotes myofibroblast differentiation and ECM accumulation [192, 193]. Conversely, fibroblasts with impaired autophagy exacerbate AEC senescence and apoptosis via altered epithelial–fibroblast crosstalk, establishing a self‐perpetuating cycle of injury and defective repair. Other cell types further modulate this network. Excessive autophagy in macrophages can drive fibroblast activation and indirectly trigger AEC apoptosis through the secretion of inflammatory cytokines. ECs, in turn, undergo autophagy‐mediated endothelial–mesenchymal transition (EndMT), analogous to EMT in AECs, contributing to vascular remodeling during fibrosis [194]. Collectively, these intercellular dynamics highlight autophagy as a central regulator of apoptosis and lung tissue homeostasis.

### 8.3 Necroptosis and Apoptosis

In IPF, apoptosis and necroptosis predominantly occur in AECs, particularly AEC2s. The injury and depletion of these cells are key drivers of fibrosis. Fibroblasts and myofibroblasts, although generally resistant to apoptosis, may undergo necroptosis under specific conditions. Immune cells, including macrophages and neutrophils, also contribute to these processes, amplifying inflammatory responses [88, 195, 196].

Within a given cell type, the balance between apoptosis and necroptosis is shaped by the integration of upstream stress signals, molecular checkpoints, genetic background, and inflammatory microenvironmental cues. In general, relatively moderate or chronic stress, such as oxidative damage or DNA injury, is more often associated with apoptotic responses, whereas more severe or unresolved stress conditions may favor necroptotic signaling [88]. Caspase‐8 functions as a critical molecular switch: when active, it drives apoptosis, whereas its inhibition shifts signaling toward necroptosis via the RIPK1‐RIPK3‐MLKL axis [196]. Genetic alterations, such as homozygous SFTPA1 (T622C), increase RIPK3 expression through the IRE1α‐JNK pathway, sensitizing cells to necroptosis [197]. Inflammatory cytokines, including TNF‐α and IFN‐γ, further potentiate necroptosis by upregulating MLKL [198]. These factors interact dynamically, enabling interconversion between cell death modalities; for instance, caspase‐8 inhibition can redirect apoptotic signaling toward necroptosis, thereby accelerating AEC loss and promoting fibrosis in IPF.

Beyond single‐cell death decisions, apoptosis and necroptosis also interact across different cell types, forming a feed‐forward circuit within the fibrotic microenvironment. Necroptotic AECs release DAMPs, such as HMGB1, which activate fibroblasts and myofibroblasts, promoting their proliferation, differentiation, and extracellular matrix deposition. In turn, fibroblasts secrete factors, including TGF‐β1, which induce AEC apoptosis, impair epithelial repair, and reinforce a self‐perpetuating cycle [199]. Necroptotic AECs additionally recruit immune cells, amplifying inflammatory responses and contributing to tissue injury [195]. Together, these intercellular interactions accelerate IPF progression and exacerbate fibrotic remodeling.

### 8.4 Pyroptosis and Apoptosis

Apoptosis and pyroptosis are functionally coupled through shared caspase signaling and gasdermin‐mediated execution, allowing apoptotic signals to be reprogrammed into pyroptotic outcomes under specific conditions and collectively promoting fibrosis [141]. Mitochondrial injury releases ROS and mtDNA, activating the NLRP3 inflammasome and caspase‐1, which cleaves GSDMD to trigger canonical pyroptosis and IL‐1β/IL‐18 maturation [200]. When GSDME is abundant, caspase‐3 cleaves it, redirecting apoptotic execution toward a pyroptotic phenotype [201]. Under hypoxia or TAK1 inhibition, TNF‐α–driven caspase‐8 can also cleave GSDMC or GSDMD, further linking apoptotic and pyroptotic pathways [202].

AECs are primary targets of these processes. Their death disrupts the epithelial barrier and releases DAMPs that recruit macrophages. Through the NLRP3–caspase‐1 axis, macrophages amplify IL‐1β–mediated inflammation, activating fibroblasts to deposit collagen and further induce epithelial apoptosis [199, 203]. The resulting barrier loss, chronic inflammation, and fibroblast‐driven remodeling drive progressive fibrosis and functional decline in IPF.

### 8.5 Other Crosstalk of Cell Death

Ferroptosis interacts with necroptosis through DAMP release and ROS accumulation, amplifying inflammation and fibrosis [204]. In cardiac fibrosis, ferroptosis also crosstalks with pyroptosis, with MLK3 acting as a shared upstream regulator. During early pressure overload, MLK3 drives inflammation and necrosis via the NF‐κB/NLRP3 pathway, while in later stages it promotes ferroptosis through JNK/p53 signaling, forming a time‐dependent cascade that accelerates fibrosis. Inhibition of MLK3 or upregulation of miR‐351 disrupts this sequence [205], underscoring the interconnected roles of ferroptosis and pyroptosis in fibrotic diseases. Ferroptosis further intersects with autophagy through nuclear receptor coactivator 4 (NCOA4)‐mediated ferritinophagy, which enhances iron‐dependent lipid peroxidation. In sepsis‐induced ALI, YAP1 suppresses NCOA4‐dependent ferritinophagy, thereby inhibiting ferroptosis and mitochondrial ROS‐mediated injury [206].

Ferroptosis and cuproptosis intersect through the TCA cycle [207, 208]. In cuproptosis, Cu^+^ mediates lipoylation of dihydrolipoamide S‐acetyltransferase (DLAT), thereby inhibiting TCA cycle activity, whereas ferroptosis relies on glutamine‐driven TCA flux to supply substrates for lipid peroxidation. Glutathione (GSH) serves as both a copper chelator and a GPX4 substrate; its depletion simultaneously triggers cuproptosis and ferroptosis, producing synergistic cell death. When GSH is exhausted, ROS generated through Cu^2+^/Cu^+^ cycling first initiate ferroptosis, while excess free copper subsequently induces cuproptosis, allowing dynamic switching between these modes. This interplay accelerates epithelial ferroptosis through the copper–ROS–GPX4 axis, impairing epithelial integrity. Meanwhile, suppression of cuproptosis‐related genes promotes fibroblast proliferation, and ferroptosis‐derived lipid peroxidation products activate TGF‐β, driving myofibroblast differentiation [209].

PANoptosis, first defined in 2019 as an inflammatory programmed cell death modality [210], involves sensors such as Z‐DNA binding protein 1 (ZBP1) and NLRP3, which assemble the PANoptosome complex. This structure integrates apoptosis (via caspases‐3/7), pyroptosis (via gasdermin D and caspases‐1/4/5), and necroptosis (via RIPK1–RIPK3–MLKL), producing a coordinated outcome that extends beyond traditional cell death pathways. In IPF, senescent or damaged AECs release DAMPs that trigger PANoptosome assembly, resulting in recurrent epithelial cell death and amplified release of proinflammatory cytokines, including IL‐1β and IL‐18. These events exacerbate chronic inflammation mediated by macrophages and neutrophils, while profibrotic factors drive fibroblast‐to‐myofibroblast differentiation and extracellular matrix deposition. Together, this cascade promotes progressive lung remodeling and irreversible fibrosis [211].

## 9 Conclusion

IPF, with its increasing incidence and poor prognosis, remains a major global health challenge. Although novel agents such as REGEND001, nerandomilast, and ifenidone have entered clinical trials, effective therapies are still limited. The initiation and progression of IPF are critically influenced by an imbalance among multiple forms of RCD. Both excessive and insufficient cell death disrupt pulmonary homeostasis, leading to amplified inflammation, aberrant fibroblast activation, and impaired tissue repair. Apoptosis, autophagy, necroptosis, pyroptosis, ferroptosis, and cuproptosis exhibit cell type‐specific alterations in AECs, fibroblasts, macrophages, and ECs, and are interconnected through complex signaling networks that collectively establish the profibrotic microenvironment. Increasing evidence suggests that these RCD programs do not operate in isolation but are coordinated through shared stress inputs, common regulatory nodes, and context‐dependent signaling dynamics, forming an integrated and adaptive cell death network. Understanding these mechanisms is essential not only for defining IPF pathobiology but also for identifying novel therapeutic targets. A key concept emerging from current research is the need for precision interventions targeting specific cell populations, regulating their survival or death to restore tissue homeostasis. Existing therapeutic strategies, whether applied individually or in combination, ultimately converge on this principle.

Emerging modes of RCD, including disulfidptosis [212], alkaliptosis [213], and lysosome‐dependent cell death [214], have recently been described. Combined with advances in single‐cell sequencing, spatial transcriptomics, and high‐dimensional flow cytometry, these findings are expected to provide deeper insights into the spatial and temporal complexity of cell death in IPF. Looking forward, integrating the crosstalk among diverse death pathways and developing more selective, cell‐targeted therapeutic strategies may open new avenues to improve patient outcomes.

## Author Contributions

X.P. and G.Y.: writing – original draft preparation, review, and editing. L.Y., M.Z., C.X. and H.L. Y.Z., Y.W., H.L., B.L.: review and editing. L.W. and G.Y.: funding acquisition. All authors have read and agreed to the published version of the manuscript.

## Funding

This work was supported by the Key R&D Program of Henan province grant 231111310400; Zhongyuan scholar 244000510009; Henan Project of Science and Technology grants GZS2023008.

## Conflicts of Interest

The authors declare no conflicts of interest.

## Data Availability

No new data were generated or analyzed in this study. All data discussed are available from the cited references.
